# Supplementary material for: Is the malaria short course for program managers, a priority for malaria control effort in Nigeria? Evidence from a qualitative study
Source: PLoS One. 2020 Jul 28;15(7):e0236576. doi: 10.1371/journal.pone.0236576 (PMC7386568; doi:10.1371/journal.pone.0236576)
Supplement: S1 File — (PDF) [file pone.0236576.s001.pdf]

NIGERIA FIELD EPIDEMIOLOGY AND LABORATORY TRAINING PROGRAM

MALARIA SHORT COURSE

NEEDS ASSESSMENT KEY INFORMANT [KII] GUIDE

Introduction
The NFELTP is conducting needs assessment towards implementing a malaria short course for program managers at different levels and other relevant stakeholders. The objective of this course is to update the knowledge of disease control program managers and other relevant stakeholders in malaria control and build their capacity on related areas. This assessment is to ensure that relevant topics and interests of stakeholders are addressed in the course curriculum. You are being asked to participate in this interview being a head of relevant department in the Ministry/development agency/NGO or director/supervisor of program manager. We acknowledge you experience in malaria control and will like you to share with us what you think are essential topics to be taught in a malaria short course especially to malaria program managers. This interview will last about 20-30mins. This interview will be audio recorded to aid complete documentation of the interview for analysis. Kindly give us some time out of your busy schedule to contribute to this laudable capacity building program.

Participants: National Coordinator National malaria Elimination Program (NMEP), thematic division Heads NMEP, Director of Public Health/Primary Health Care (PHC) and Disease Control, Executive Secretary State Primary Health Care Development Agency/Board, State Malaria Program Managers, Director PHC/PHC Coordinator, LGA RBM, Head of malaria units in donor organizations, Malaria focal persons/unit in development partners, Disease Control Programs at national, state and LGA levels.

The interviewer proceeds to informed consent section and obtain written consent.

1. Institution 2. Date
3. Name: ________________________________
4. Department or control program: ……………….
5. Designation of Key Informant…………
6. How many years as head/supervisor in this department/position: ……………..
7. Time interview started……… 8. Time interview finished…………viii.
9.Name of facilitator: __________________________
10. Name of note taker/interview recorder: ____

Malaria control in Nigeria
11. What are the current strategies for malaria control in Nigeria?
Probe points/questions for each strategy:
• What issues/problems do/does you/your team encounter while implementing this strategy?
• How do/does you/your team address those issues/problems?
• What additional knowledge/skills will you/your team require/s to effectively implement the strategy?

12. We know that each organization plans for their program activities periodically including malaria. Could you share with us the process?

Probes

- Who are those involved in the planning process? Are your program managers involved in planning?
- What additional knowledge and skills do you think your program manager require for better planning?

Malaria Policy

13. Nigeria has a NMEP; what is your opinion about achieving the policy goals/target?
Probe

- What are the challenges to implementing policy? What are the biggest gaps in terms of skills to implement policies effectively?
- How will a malaria short course contribute to successful implementation of malaria control policy?
- What are additional knowledge and skills do you think can be harnessed for policy implementation?

Surveillance and supervision

14. Please share the process of malaria surveillance in your LGA/state/national program.
Probing points/questions:

- Can you describe the reporting system in your LGA/state/national program (monthly, quarterly, and yearly information system reports)?
- Are these reports discussed routinely within your program? (If not, why not?)
- How do you think this malaria short course can help to improve the quality of data generated at different levels of LGA/state/national program?

15. What should be the focus of training to reduce challenges and improve skills of program managers to interpret and utilize malaria data from the different levels of collection?

16. What additional knowledge and skills do you think program managers require to supervise your program more effectively?

Comprehensive Capacity building

Personnel are posted from one control program to the other within the LGA/state/national and may eventually be posted to the malaria program.

17. What plans do this organization have to orientate the newly posted personnel to your program?
Probe:

- What are the knowledge and skills that you think they need to acquire to function effectively as malaria program manager?

Personnel training and Support for malaria short course

18. What is your policy on continuing education for your staff in your LGA/state/national program?

- Probe (about inclusion of training fund in yearly budget)

19. How will the release of your program officer for the duration of the short course, have an impact on your program?
[This include a total of 11–12 days in learning workshops, interspersed with 8 weeks on-the-job fieldwork with supervision at the trainees' place of work]

20. How will you support the program officer to implement the on-the-job field assignment?
11. What plans (financial, moral, material, etc.) do you have to support your program officers to attend this short course annually?
21. ***Thematic Heads in NMEP/SMEP.*** Could you share specific skills or knowledge that is required in your unit that this course should address?

Dissemination and utilization of information

22. Describe the process of communication between the program managers and the management (include the management process and how the organization/program utilize the information)

Public Private Partnership in malaria control
23. How can we build Public-private partnership for the sustainability and enhanced participation in this course?
Probe:
How can we engage partners to support capacity building of staff at the LGA/State/National?

Is there anything else you would like to add regarding the utility of a malaria short course for malaria program manager and other program officers in Nigeria?

Thank you.

INFORMED CONSENT FORM

IRB Research Approval Number: UI/EC/18/0089

This Approval will elapse on: 21/06/2018

Title of research:

NEEDS ASSESSMENT FOR MALARIA SHORT COURSE IN NIGERIA

Name and affiliation of researcher:

This study is being conducted by Nigeria Field Epidemiology and Laboratory Training program This will be led by Dr IkeOluwapo O. Ajayi of the Department of Epidemiology and Medical Statistics, College of Medicine, University of Ibadan. Should you have any questions or concern about this study please contact Dr IkeOluwapo O. Ajayi on Tel: 08023268432.

Purpose of research:

The purpose of this research is to identify the knowledge/skills needed to provide the necessary services needed for malaria elimination in Nigeria.

Procedure of the research, what shall be required of participants:

Participants will be expected to complete a questionnaire, which consists of 4 sections: general information, malaria workshops attended in the last five years, Job description and needs in malaria and its control. In addition, some participants will participate in interviews.

Participants will be expected to share experiences and provide opinions during key informant interviews.

Expected duration of research and participant(s) involvement:

The needs assessment exercise will cover a period of about three months. The participant will spend approximately 20 to 30 minutes to complete the questionnaires and 30 – 40 minutes for key informant interviews.

Risk(s):

It is expected that this research would pose no physical, biological or social harm to all the research participants as all the procedures involved are non-invasive and no samples (blood, urine, saliva) will collected.

Cost of Participating, if any, of joining the research:

Your participating in this research will cost you nothing financially but your time and effort in participating

Benefit(s):

The goal of this research is towards identifying the areas of imminent knowledge and skills lacking among malaria project managers but are needed for effective and efficient delivery of services towards control and eventual eradication of malaria in Nigeria.

Confidentiality:

We will maintain complete confidentiality and anonymity of your responses. Data would be protected in a password protected computer. As part of our responsibility to conduct this research properly, officials from the UI/UCH Institutional Review Board may have access to these records.

Voluntariness:

Your participation in this research is entirely voluntary

Due inducements

You will not be paid fees for participating in this research

Consequences of participant's decision to withdraw from research:

You can also choose to withdraw from the research at any time. However, we hope that you will participate since your views are important.

Statement of person giving Consent:

I have read the description of the research. I understand that my participation in this research is voluntary. I know enough about the purpose, methods, risk and benefits of the research study to judge that I want to participate in it. I understand that I may freely stop being a part of the study at any time. I have received a copy of the consent form and additional information sheet to keep for myself.

DATE_________________________SIGNATURE___________________________

NAME____

Detailed contact information including contact address, telephone, fax, e-mail and any other contact information of researcher(s), institutional HREC and Head of Institution:

This research has been approved by the UI/UCH Ethics Committee and the chairman of this committee can be contacted 08033264593 for further information/questions.

PLEASE KEEP A COPY OF THE SIGNED INFORMED CONSENT.
