# Supplementary material for: Is the malaria short course for program managers, a priority for malaria control effort in Nigeria? Evidence from a qualitative study
Source: PLoS One. 2020 Jul 28;15(7):e0236576. doi: 10.1371/journal.pone.0236576 (PMC7386568; doi:10.1371/journal.pone.0236576)
Supplement: S2 File — (PDF) [file pone.0236576.s002.pdf]

Development and implementation of a malaria short course: A capacity building effort towards achieving malaria elimination in Nigeria. May 2017 – March 2019

Participating organizations

Department of Epidemiology and Medical Statistics, College of Medicine, University of Ibadan,
Nigeria Field Epidemiology and Laboratory Training Program
African Field Epidemiology Network (AFENET)
President's Malaria Initiative (PMI)
National Malaria Elimination Program, Federal Ministry of Health, Abuja

Description of the course and the outcome

With technical support from Presidents Malaria Initiative (PMI) and National Malaria Elimination Program (NMEP) the Nigeria Field Epidemiology and Laboratory Training Program (NFELTP) designed and implemented the first national malaria short course training program. This is to strengthen the capacity of program managers and other stakeholders especially those in malaria control programs to effectively oversee the malaria control activities in the state and support the implementers of malaria treatment guidelines to provide quality service and adhere to guidelines.

NFELTP and PMI constituted a team for the development of a curriculum for the course. A concept note was developed and presented to PMI for approval. The first draft of the curriculum was developed using information from the literature and borrowing idea from the existing NFELTP Frontline Short Course. A needs assessment survey (NAS) was conducted among stakeholders and thereafter the draft curriculum was revised using NAS findings. A consensus consultative meeting with key stakeholders was held to further revise the curriculum. The revised draft was shared with the NMEP, PMI and CDC experts for face validation. The draft was revised severally before the final draft curriculum was agreed upon. The curriculum comprises 20 modules (workshop 1), 17 (workshop 2) and 3 (workshop 3). Curriculum includes group projects and field work assignments to provide practical experience in data quality audit, data analysis, report writing, monitoring and surveillance, problem analysis, advocacy and logistics management.

The first cohort training was held November 2018 to March 2019 with 29 participants from 6 states and the FCT. Nine participants were females and 67% were from PMI-supported states. Over 95% judged the course to be excellent/good. Overall, mean score was 8.61/10.0, content, 8.72; quality of instruction, 8.27; and participant materials, 8.5. Lectures and presentations received an average rating of 8.67.

Malaria short course is carried out over a 3-month period, and consists of three workshops, separated by periods of field work (on-the-job projects).

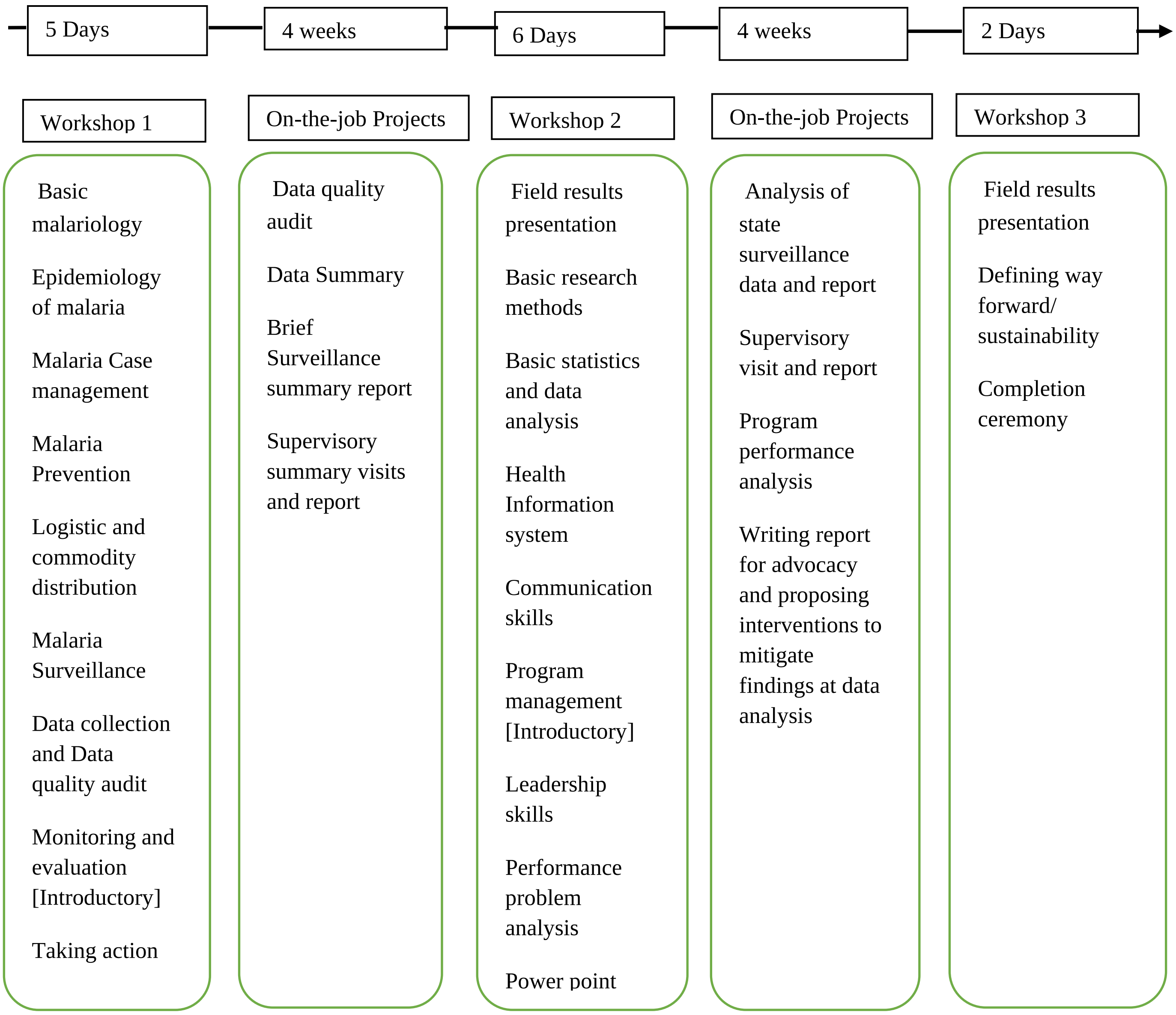

Figure: Diagrammatic representation of the Malaria Short Course
