# Supplementary material for: Is the malaria short course for program managers, a priority for malaria control effort in Nigeria? Evidence from a qualitative study
Source: PLoS One. 2020 Jul 28;15(7):e0236576. doi: 10.1371/journal.pone.0236576 (PMC7386568; doi:10.1371/journal.pone.0236576)
Supplement: Attachment — Submitted filename: Response to reviewers May, 2020.pdf [file pone.0236576.s004.pdf]

May 29, 2020

The Editor,

PlosONE

Dear Editor,

This is to acknowledge the effort of the editorial team for their immense contributions towards transforming the manuscript to its present form. All the observations raised, and the corrections and comments made by the reviewers are appreciated and have been addressed.

Moreover, point—by-point response to reviewers'/editorial comments have been addressed as described in the appendix. The page and line numbers refer to those in unmarked version of the revised manuscript tagged "Manuscript_clean_May 2020". The dataset can be accessed at https://zenodo.org/record/3588514#.XpT2VMhKjIU

Should you require additional information or if you have any questions, please contact me. I will be pleased to hear from you.

Yours sincerely,

Olufemi Ajumobi (Corresponding author)

University of Nevada, Reno, USA

femiajumobi@gmail.com

Section	Editor/Reviewers' comments	Authors response
**Editor**		
Abstract	P4: Abstract/Conclusion-It is better to cut "The first cohot of….trained on MSC." This sentence is not related with the results.	Thanks. This has been deleted from the concluding section of the abstract.
Body of manuscript	P7: "Materials and methods" should be "Methods"	Thanks. This has been revised accordingly.
	P8: and other pages: Please avoid abbreviations which are not familiar with international readers: LGA, NMEP, NAVRC, SMEPs, etc. There are many others.	Thanks. All relevant abbreviations have been defined at first use.
	P9: Please add a short description of the malaria short course as an appendix.	Thanks. This has been provided as S2 file under the supporting information.<br>See page 29, line 3
	P13L2: Please be more specific about under-qualified ones. Are they community health volunteers?	This refers to community volunteers and has been replaced accordingly, see page 13, line 3
	P21: "All the key informants" mean 56 professionals? Stakeholders, professionals, key informants…these terms are interchangeably used?	Thanks. This has been revised. The study was conducted among malaria stakeholders (key informants) who were also professionals. The aim of the training curriculum is to build capacity of program managers. This distinction has been made in the revised manuscript.<br>See page 11 line 15<br>See page 8, line 14
	P22: It is better to show a summary of major findings in the first paragraph of discussion section, with no reference, no discussion. From the second paragraph, discussion of each major finding may start.	Thanks. This has been addressed.<br>See page 18, lines 19-26<br>See page 19, lines 1-4
**Reviewer #1**		
Background		
	1) The authors did not include any checklists for qualitative research such as COREQ or SRQR.	This is noted, thanks. COREQ checklist has been included.

Methods	2) A more detailed sampling strategy and inclusion/exclusion criteria must be included. For example, under the "Data Collection" subheading, the authors mentioned that "the interviews were conducted among 60 purposively selected stakeholders". However, in the results, the authors reported "the participants were 56 professionals". What happened to the other 4 stakeholders?	Thanks. The mix-up/error has been corrected. The key informant interviewees were 56 and not 60. Initially we desired to have 60 interviewees, but we concluded on 56 interviews when saturation was achieved.<br>See page 8, lines 9-14<br>See page 9, line 22
	3) In "Data processing and analysis", in what language were the interviews conducted?	Thanks. The interviews were conducted in English language.<br>See page 9, line 7
Results	4) The presentation of Table 1 could be improved.	Thanks. This has been addressed (an abridged title has been used and redundant texts were removed).
Discussion	5) In the discussion, the authors did not discuss any limitations or the potential sources of bias at all. Please discuss the limitations and the potential sources of bias as this will strengthen the scientific value of the paper.	Thanks. This has been addressed at concluding part of the discussion.<br><br>See page 22, lines 22 – 24<br>See page 23, lines 1-2
Conclusion	6) The title is a little confusing. The title only mentioned "program managers" but in the discussion and conclusion, the authors also mentioned "relevant health workers".	This has been addressed. Though program managers are also health workers. For consistency, program managers were retained in the discussion and conclusion sections.<br>See page 1, lines 1-2.
Others Minor issues	7) Please include line numbers in the manuscript for easier review.	Noted and addressed, thanks.
	8) The authors used "6.2 ± 4.7 years". Is this an appropriate way to present standard deviation?	This is a recognized way to present the mean and its standard deviation. Moreover, the authors have used another recognized approach which states standard deviation explicitly (….6.2 (standard deviation: 4.7 years).<br><br>See page 12, lines 1 -2
	9) I suggest that the whole manuscript be sent for scientific editing. For example, there are many minor typo errors.<br>λ In Page 5, line 3, "However, there 217 and 219 million malaria cases…"	Thanks. The manuscript has been edited appropriately. Minor errors (including typographical ones) have been corrected in the body of the manuscript and references.

	λ Page 5, line 13, "…certified to be malaria-free by WHO [5])."<br>λ Page 6, the authors used "%" and "percent" interchangeably. Please be consistent.<br>λ Please check the reference for typo errors. For example, reference number 7, "World Healthj Organization…"	"percent" has been replaced with "%" for consistency<br><br>The relevant section in introduction has been revised. See page 5, lines 5-6
	**Journal requirement**	
	1. Please ensure that your manuscript meets PLOS ONE's style requirements, including those for file naming	Thanks. This has been addressed.
	2. Please include additional information regarding the interview guide used in the study and ensure that you have provided sufficient details that others could replicate the analyses. For instance, if you developed a guide as part of this study and it is not under a copyright more restrictive than CC-BY, please include a copy, in both the original language and English, as Supporting Information. In addition, please provide details of any pre-testing of this guide that took place.<br><br>- Please ensure you have thoroughly discussed any potential limitations of this study within the Discussion section, for example the potential bias introduced when using qualitative data and the skewed nature of the dataset.	Thanks. The interview guide was originally designed by the researcher and pretested, See page 8, line 19. Sufficient details have been provided (See page 8, lines 8 -22; page 9, lines 1-22.) and the guide included as supplement file S1 under supporting information (See page 29, line 2).<br><br>Thanks. Limitations have been included at the concluding part of the discussion.<br><br>See page 22, lines 22 – 24<br>See page 23, lines 1-2
	3. During your revisions, please confirm whether the wording in the title is correct and update it in the manuscript file and online submission information if needed. Specifically, ensure that the title is grammatically correct.	Thanks. The title is correct and has been edited with addition of 'the': "Is the malaria short course for program managers, a priority for malaria control effort in Nigeria? Evidence from a qualitative study"<br>See page 1, line 1
	4. In your Data Availability statement, you have not specified where the minimal data set underlying the results described in your manuscript can be found.<br>please upload your study's minimal underlying data set as either Supporting Information files or to a stable, public repository and include the relevant URLs, DOIs, or accession numbers within your revised cover letter.<br>We will update your Data Availability statement to reflect the information you	This has been reflected in the cover letter (response to reviewers) and can be found at https://zenodo.org/record/3588514#.XpT2VMhKjIU (Supporting information, S3).<br><br>See page 29, line 4

	provide in your cover letter.	
	5. One of the noted authors is a group or consortium: NFELTP fellows. In addition to naming the author group, please list the individual authors and affiliations within this group in the acknowledgments section of your manuscript. Please also indicate clearly a lead author for this group along with a contact email address.	The individual authors were listed earlier in acknowledgements section. The name and email of the lead in the author group have been included in the authorship page (See pages 1 - 2).
	6. We note you have included a table to which you do not refer in the text of your manuscript. Please ensure that you refer to Table 1 in your text; if accepted, production will need this reference to link the reader to the Table.	Thanks. This has been addressed. See page 10, line 2.
**Reviewer #2**		
Abstract	Not clear if the study was done after the training or before the training	The study, a need assessment, was conducted prior to the implementation of the training, the Malaria Short Course.<br><br>This has been reflected with clarity in the abstract.<br>"Prior to its implementation, a needs assessment was conducted from the perspectives of stakeholders to plan and develop the curriculum".<br>See page 3, lines 5 - 6<br><br>And in the introduction<br>"This study was designed as a need assessment from the perspectives of stakeholders for planning and developing curriculum for a comprehensive malaria course short (MSC) which seeks to build capacity of program managers for malaria control in Nigeria"<br>See page 6, lines 14-17
	Study design is missing	This has been included. "This was an exploratory qualitative study."<br>See page 3, line 9<br>See page 8, line 3

	Sampling missing (even the size is missing) though not common for qualitative study, but at least observation to the "Principal of saturation" is essential	56 interviews were done.<br>See page 3, line 9<br>See page 8, line 14<br>Saturation was attained, as stated in the body of the manuscript,<br>See page 9 line 22.
	Development of tools and content thereof	The contents of the data collection instrument were stated and the sentence has been edited.<br>"Opinions on the need for training, its perceived impact, priority focus, likelihood of participation, sustainability of and planned support for the MSC were explored using a pretested researcher-designed interview guide."<br>see page 3, lines 10-12.
	Analysis plan and theory involved	The analysis was not based on a theory. The data analysis method has been included. "..and the transcripts were subjected to thematic content analysis."<br>See page 3, line 13
	Conclusion: issues with payment for training as explained above	Thanks. This is in tandem with the results in the abstract and the body of the manuscript.<br>See page 4, line 6
Background	Too broad and not focused to the problem at hand. The focus needs to be narrowed to high level workers (Program managers) and rationale for this.	Thanks. This has been refocused. Irrelevant texts were deleted.<br>See page 5, lines 1 -22.<br>See page 6, lines 1 -22.<br>See page 7, lines 1 -2.
	Clear problem is missing and implication thereof	Thanks. The initial statement of problem has revised with addition of implications. "The high turnover rate of program managers especially at the state level and non-provision of training for incoming officers are the major contributors to this, resulting in proliferation of newly appointed program managers bereft of technical and programmatic skills for malaria control."

		See page 5, lines 15 - 19.
	Clear research questions are missing and therefore SMART objectives	The main of the study has been edited for clarity and specific objectives have been stated. "This study was designed as a need assessment from the perspectives of stakeholders for planning and developing curriculum for a comprehensive malaria course short (MSC) which seeks to build capacity of program managers for malaria control in Nigeria. The specific objectives of the study were (1) to assess stakeholders' opinions relating to need for a Malaria Short Course (MSC) for program managers; (2) to describe how the MSC can help improve the quality of data generated at the local government area (LGA) and state programs; (3) to identify priority focus of training that would improve skills of program managers for effective data utilization and skills required to supervise program effectively; (4) to determine thematic areas to consider for the MSC; to assess willingness of organization to release staff for the MSC; and (5) to determine the sustainability of and planned support for the MSC."<br>See page 6, lines 17 - 22<br>See page 7, lines 1-2<br><br>The 'MART' aspect of SMART objectives does not apply. These are used usually used for deliverables in the context of programmatic/project implementation, which was not the case in our study.
Methods	Study design is not mentioned. In the section responsible for this, it was mentioned "an explorative study". Explorative studies do not specify study design.	Thanks. This has been addressed. The design was exploratory qualitative study.<br>See page 8, line 2
	2. The analysis plan lack theoretical framework for the qualitative analysis. It would introduce and describe the theory that explains the "why" in the research problem.	Thanks. This was not a theory driven research, unlike the case for some social behavioral science studies. This utilized Qualitative description 'QD', a well-recognized approach

		which aims to present a rich, straight description of an experience or an event without a theoretical underpinning. Data was analyzed using Thematic content analysis. The study was conducted, as a needs assessment for the training curriculum on malaria through the exploration of opinions and experiences of the study participants
	3. Both introduction and discussion are general. There is lack of focus in the specific study and context thereof. These sections need to be focused into malaria in the context of the leaders giving the strategic direction/leadership in malaria program, rather than the burden and epidemiology thereof. Only the last two paragraphs speak of this	Thanks. The context of introduction and discussion have been revised to reflect program managers, who provide leadership and strategic direction at local, state and national levels. The content on burden and epidemiology has been revised appropriately with an abridged and focused introduction section.<br>See page 5, lines 1 -22<br>See page 6, lines 1 -22<br>See page 7, lines 1 -2<br>See page 18, lines 19-26<br>See page 19, lines 1-23<br>See page 20, lines 1-22<br>See page 21, lines 1-24<br>See page 22, lines 1-24<br>See page 23, lines 1-5
	4. The conclusion is somehow inflated. I have gone through the evidence presented, and could not see where the PM were reluctant to provide funding/scholarships for their employees'	The conclusion is grounded in the findings. Please see below quotes (See page 17, lines 15 - 26)<br>*We do have funds for those that are going for in service training. Government is sponsoring them and paying them their salary but for these short courses we do not have budget for that (Participant 16, Director of PHC)*<br><br>*No training fund, I mean at the LGA level but though at the state we do have trainings. The ones we do at the LGA level is purely our personal sacrifice*

		(Participant 3, Director of PHC).
	Re-write selection (convenience) and reasons for selection of interviewee	Thanks. This has been revised. The sentence "Participants were selected across LGA level, state and Federal Ministries of Health and non-governmental organizations for holistic views and representativeness" was added.<br>See page 8, lines 12 – 14)
	Data collection needs to be expanded to also include how the questions were asked, preps of the RAs (if used), training, pre-test (where, how), and people involved in interview, notes taking etc.	Thanks. We have provided extensive information about data collection including pre-test of the instrument, data collection process (including open-ended nature of the questions) and training of the research assistants**.**<br>See page 8, lines 8 – 22<br>See page 9, lines 1 - 22
	Explain about the tool, development, and content thereof	Thanks. This has been addressed in the revised content of data collection section
	The analysis plan lack theoretical framework for the qualitative analysis. It would introduce and describe the theory that explains the "why" in the research problem.	Thanks. This was not a theory driven research, unlike the case for some social behavioral science studies. This utilized Qualitative description 'QD', a well-recognized approach which aims to present a rich, straight description of an experience or an event without a theoretical underpinning. The study was conducted, as a needs assessment for the training curriculum on malaria through the exploration of opinions and experiences of the study participants. Data was analyzed using thematic content analysis.
	Ethical consideration needs to be	A section on ethical consideration has been included.<br>See page 11, lines 4 - 11
Results		
Discussion	Focus on the context	Thanks. This has been addressed as per the earlier comments.

	Add recommendations	Thanks. Recommendations have been reflected in the conclusion section.<br>See page 23, lines 15 - 21
Conclusion	Conclusion on payment/support has no results attached	Thanks. This is in tandem with the results in the body of the manuscript.<br>See page 17, lines 15 - 26
Reviewer #3		
Abstract		
Body of manuscript	It is not clear a definition of “short” or “long” for “Malaria Short Course”. It is better to clarify the definition of “short” in this context.	This has been addressed.<br>See page 5, lines 20 – 22
Background	In Page 15, Comment of a Participant 42, M&E officer<br>“HMIS” should be spelled out “Health Management Information System (HMIS)” since this word appeared first time.	Thanks. This has been addressed.<br>See page 15, line 28.
Results	Expressions, especially in the Results section, seemed redundancy. I recommend the authors to revise the texts to avoid redundancy and make them concise. Some description in the results and discussions were also partially overlapped.	Repeated and redundant statements and quotations were removed from the manuscript in order to improve the manuscript and make it more concise and focused.
	The authors described “Malaria Short Course” and “malaria short course”. This should be uniformed.	Thanks. This has been addressed.
	4. In Page 19, Comment of a Participant 48, State Malaria M&E officer<br>Please edit “Microsoft excel and power-point” to “Microsoft Excel and Power Point”.	We have changed “Microsoft excel and power-point” to “Microsoft Excel and Power Point”.<br>See page 16, lines 21-22
Conclusion		
	It is better to summarize comments and challenges obtained from the study participants in a table.	Thanks for your comments. Moreover, the authors preferred presenting these in prose for ease of comprehension and readability
	Grammatical errors, such as, singular & plural should be corrected.	Thanks. The manuscript has been perused and edited appropriately.
