# Supplementary material for: Is the malaria short course for program managers, a priority for malaria control effort in Nigeria? Evidence from a qualitative study
Source: PLoS One. 2020 Jul 28;15(7):e0236576. doi: 10.1371/journal.pone.0236576 (PMC7386568; doi:10.1371/journal.pone.0236576)
Supplement: Attachment — Submitted filename: Response to reviewers.docx [file pone.0236576.s005.docx]

July 5, 2020

The Editor,

PlosONE

Dear Editor,

This is to acknowledge the effort of the editorial team for their immense contributions towards transforming the manuscript to its present form. All the observations raised, and the corrections and comments made by the reviewers are appreciated and have been addressed.

Moreover, point—by-point response to reviewers’/editorial comments have been addressed as described in the appendix.

Should you require additional information or if you have any questions, please contact me. I will be pleased to hear from you.

Yours sincerely,

Olufemi Ajumobi (Corresponding author)

University of Nevada, Reno, USA

[femiajumobi@gmail.com](mailto:femiajumobi@gmail.com)

| **Section** | **Editor/Reviewers’ comments** | **Authors response** |
| --- | --- | --- |
| **Editor** | | |
|  | 1. P3 L18: Please do not use ± in academic writing. 6.2 (SD 4.7) is better. See below site: <https://www.ncbi.nlm.nih.gov/pmc/articles/PMC2959222/#CIT2> | Thanks. This has been addressed |
|  | 2. P4 L13: Are these all MeSH key words? It is better to use MeSH keywords as much as possible to be quoted in the future. | These keywords ‘Malaria, capacity building, curriculum, training needs assessment, program managers, short course’ were replaced with ‘Malaria, capacity building, curriculum, needs assessment, program, administrative personnel’ available in MeSH |
|  | 3. P6 L20: LGA is used in here as abbreviation, and then in P7 L9. As LGA is not a common abbreviation for international leaders, it is better to spell it out always. | Thanks. This has been addressed |
|  | 4. P6 L18-P7 L2: Five objectives are written in here. In the results, some of these five objectives are not well addressed as major objectives. It is better to modify either these objectives or results (subheadings), so that the objectives and results can be well matched. | We have revised the objectives to be 3 and merged those that are not well addressed as an objective under a relevant major objective  In the results section, we have revised headings of the sub sections to reflect the 3 objectives.  1) to assess stakeholders’ opinions on the need for a MSC for program managers  (2) to identify priority focus of training and thematic areas that would improve skills of program managers for effective discharge of their duties including supervision skills  (3) to determine the sustainability of, and planned support for MSC, and willingness of stakeholders’ organizations to release staff for the MSC |
|  | 5. P8 L2: FCT should be always spelled out. | Thanks. This has been addressed |
|  | 6. P8 L14, L9: Key informant interview is abbreviated as KII twice. As KII is not frequently used, always spell it out. | Thanks. This has been addressed |
|  | 7. P9 L6 and many other places: Please use male and female only as adjectives. It is better to use men and women. Male assistants are fine. | Thanks. This has been addressed |
|  | 8. P10: One paragraph is too long. One paragraph should have only one message. L14 may be a place to start a new paragraph. Please check other long paragraphs, too. | The paragraph has been broken into shorter one as advised. Other long paragraphs have been broken as appropriate. |
|  | 9. P12 Table 1. As the number is only 56, percentage may not be necessary in this table. | Thanks. This has been addressed |
|  | 10. P18 L20: MSC is forgotten. | Thanks. This has been addressed |
|  | 11. P22 L22: Usually qualitative research is not conducted for generalization. Is this statement based on some current books or academic papers? If so, please quote it. | No, qualitative research is not conducted for generalization. Reference has been inserted ‘Fossey E, Harvey C, McDermott F, Davidson L. Understanding and evaluating qualitative research. Aust N Z J Psychiatry. 2002;36(6):717-732. doi:10.1046/j.1440-1614.2002.01100.x |
|  | 12. P26~: Reference section is not professionally done. Sometimes journal names are abbreviated, sometimes they are fully spelled out. | Thanks. References are fully spelled out. |
| **Reviewer #1** | | |
| Background | 1) Page 5 Line 4: high Impact -> High Impact? (Capitalize the ‘H’?) | Thanks. This has been addressed |
|  | 2) Page 6 Line 4: The United President’s -> The United States President’s? | Thanks. This has been addressed |
|  | 3) COREQ Checklist was not submitted as an attachment? | Yes, COREQ checklist was submitted. |
|  | 4) Page 10 Line 5: Please check the grammar of this sentence: “further validated by the study experienced qualitative data” | Thanks. This has been addressed |
|  | 5) Page 10 Lines 16-17: Grammar: “Following Nowell et al’s step by step approach to thematic analysis.” Not a complete sentence. | Thanks. This has been addressed |
|  | 6) Page 22 Lines 14-20: Private-public partnership is important and recommended by the WHO. If possible, include a citation and a one-sentence description of the example of the effectiveness of private-public partnership in other similar settings, if there is any. | Thanks. The sentence was retained as it was. |
|  | 7) Page 22 Line 24: “Generalizability is not the goal” -> This sentence is redundant. | Thanks. The sentence was deleted. |
| **Reviewer #3** | | |
|  | In the introduction, the authors mentioned that “The term “short course” entails a training delivered for short duration (four to five days) interspersed by period of practicum/field assignments,”…. However, Supporting Information S2 shows that “Malaria short course is carried out over a 3-month period”. The workshop is 2 to 6 days, but on-the-job project is 4 weeks. Therefore, the description in the Introduction should be edited. | Thanks. This has been addressed |
|  | I suggest to the authors that the manuscript should be checked by an English editor because there are still many grammatical errors. For example, article (the) is missing in the key words, such as “MSC”, “LGA”. | Thanks. This has been addressed |
| Other minors | Description should be consistency. In page 6, line 16 in Introduction: Please correct “malaria course short (MSC)” to “malaria short course (MSC)”. In page 18, line 20 in Discussion: Please correct “the short malaria course” to “the MSC”. | Thanks. This has been addressed |
|  | In page 9, line 13 in Methods: Please correct “the researcher assistants” to “the research assistants”. | Thanks. This has been addressed |
|  | Only once would be enough to describe full spell with abbreviation. In page 6, line 18 in Introduction: Please correct “malaria short course (MSC)” to “MSC”. | Thanks. This has been addressed |
|  | In page 7, line 8 in Methods: Please correct “Local Government Areas (LGAs)” to LGAs. | Thanks. This has been addressed in line with reviewer 1 comments not to use LGA being an uncommon abbreviation |
|  | In page 18, Line 25 in Discussion: Please correct “Africa countries” to “African countries”. | Thanks. This has been addressed |
|  | In page 19, line 11-12 in Discussion: Please correct “computer and information technology skills and were also identified.” to “computer and information technology skills were also identified.” | Thanks. This has been addressed |
